# Construction Strategy and Mechanism of a Novel Wood Preservative with Excellent Antifungal Effects

**DOI:** 10.3390/molecules29051013

**Published:** 2024-02-26

**Authors:** Lei Wang, Teng Wang, Ruidi Hao, Yamei Wang

**Affiliations:** 1 College of Materials Science and Art Design, Inner Mongolia Agricultural University, Hohhot 010018, China; wanglei792@163.com (L.W.); forest20231227@163.com (R.H.); 2 Inner Mongolia Key Laboratory of Sandy Shrubs Fibrosis and Energy Development and Utilization, Hohhot 010018, China

**Keywords:** environmentally friendly, wood preservatives, biodegradation, antifungal property

## Abstract

Wood is a naturally porous material prone to microbial erosion and degradation in outdoor environments. Therefore, the development of an environmentally friendly wood preservative with excellent antibacterial effects and low toxicity is urgently needed. In this study, nitrogen-doped carbon quantum dots (N-CQDs) with excellent antifungal performance and fluorescent properties were synthesized using a one-step hydrothermal method with chitosan quaternary ammonium salt (HACC) as the raw material. The fluorescence characteristics of N-CQD preservatives can help track their position and distribution in wood. The minimum inhibitory concentration (MIC) of N-CQDs is 1.8 mg/mL, which was nearly 22 times lower than that of HACC (40.0 mg/mL) in the PDA medium. The decay resistance test demonstrated that wood treated with N-CQDs showed a considerably reduced decay degree and its mass loss rate decreased from 46 ± 0.5% to 3.8 ± 0.5%. Biological transmission electron microscopy revealed that N-CQDs effectively destroyed fungal cell structures, thereby hindering the growth of *Coriolus versicolor*. N-CQDs synthesized using the one-step hydrothermal method can be used as an efficient wood preservative that can effectively improve the utilization and service life of wood.

## 1. Introduction

Wood is widely used in daily life owing to its properties such as recyclability, renewability, high mechanical strength, low density, and insulation [1]. However, the chemical components of wood are primarily lignin, hemicellulose, and cellulose, which are easily degraded by micro-organisms, such as fungi and termites, under suitable temperature and humidity conditions [2,3]. Previous studies have shown that wood treated with preservatives has a prolonged life; however, chemical preservatives, such as alkaline copper quaternary and copper chromium arsenic, remain the most commonly used wood preservatives [4]. Chemical preservatives have certain toxic effects and can cause harm to the environment, humans, and animals [5]. Therefore, finding green and efficient wood preservatives with low toxicity is a major issue that needs to be urgently addressed in the field of wood preservation [6].

Chitosan quaternary ammonium salt (HACC) is a biobased material derived from marine-organism chitosan [7]. HACC provides environmental protection and has low toxicity, as well as anticancer, antibacterial, and other pharmacological effects [8,9,10]. HACC with antibacterial properties has recently been used in sustained drug release and biomedical applications [11,12]. However, research on its wood protection effects and antifungal performance is scant. *Coriolus versicolor* is a common decay fungus with a strong ability to degrade wood. Exploring the antifungal effects of HACC in the field of wood protection is of great importance. Current research indicates that appropriate nanomodification methods can be used to improve the antibacterial performance of HACC, thereby expanding its application scope [13,14].

Carbon quantum dots (CQDs) are novel fluorescent nanomaterials with particle sizes of less than 10 nm. They contain abundant carbon atoms, which can generate carbon skeletons and further form carbon nuclei through chemical reactions [15,16]. Various materials for CQD synthesis exist. They include inorganic carbon sources, such as carbon nanotubes and carbon fibers, and organic carbon sources, such as biomass materials [17,18,19]. CQDs are often oblate in shape and their dispersibility is good, and they have attracted widespread attention from scholars owing to their unique physical and chemical properties [20,21]. Surface functionalization modification is the main factor determining these performances (such as optical, electrical, and chemical performance) [22,23]. The modification of the surface functional groups of CQDs can adjust material properties to meet the needs of practical applications [24]. Previous studies conducted different functionalization treatments on CQDs by introducing heteroatoms, such as N, S, and P, for atomic doping during synthesis [25,26,27]. After atomic doping, the surface groups of CQDs are more abundant and have better water solubility [28], fluorescence [29], and antibacterial properties [30]. CQDs are commonly used in fields such as drug delivery [31,32], tissue engineering [33,34], fluorescence labeling [35,36], and food preservation [37,38].

In this study, an efficient N-CQDs wood preservative with low toxicity was prepared through nitrogen doping using HACC as the carbon source. The results indicate that N-CQDs have excellent fluorescence performance. The position and distribution of N-CQDs impregnated into wood can be tracked based on their fluorescence, enabling visualization. Moreover, the antifungal effects of HACC and N-CQDs were compared. HACC prepared into N-CQDs showed considerably improved antifungal performance and reduced the cost of preservatives. Meanwhile, the antifungal mechanism of N-CQDs against *C. versicolor* was elucidated through transmission electron microscopy. The N-CQDs synthesized in this study are an effective wood preservative and provide a reference for the future application of nanowood preservatives.

## 2. Results and Discussion

**Figure 1:**
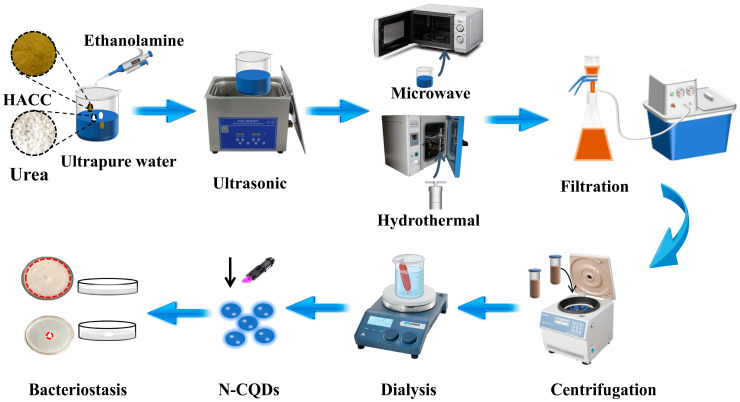
Flowchart of N-CQDs prepared using two different methods (microwave and hydrothermal methods).

The schematic diagram of N-CQD synthesis is shown in Figure 1. In this study, HACC was used as the precursor of the reaction, and urea and ethanolamine were used as nitrogen dopants together. N-CQDs with excellent fluorescence were synthesized using microwave and hydrothermal methods. The optimal preparation method for N-CQDs was identified based on the antifungal effect on the PDA culture medium.

### 2.1. Selection of Modification Methods

The antifungal effect of CQDs is related to their positive surface charges, modified functional groups, and nanoparticle size [39]. Moreover, it can be adjusted by doping with different types of nitrogen-containing functional groups. The antifungal activity of N-CQDs can also be influenced using the synthesis methods [40]. Therefore, the antifungal activity of HACC after nanomodification using different synthesis methods and the addition of nitrogen dopants was evaluated. Among the N-CQDs synthesized using different methods, those synthesized using the hydrothermal method had the best antifungal effect. Among the nitrogen admixtures, urea (CH_4_N_2_O) and ethanolamine (C_2_H_7_NO) had the strongest antibacterial effect when used together.

The most effective N-CQD modification method was shown in Figure S1. The antifungal effect of N-CQDs synthesized using different methods was evaluated based on the diameter of the fungal colony. Compared to the blank group (g), N-CQDs synthesized using microwave and hydrothermal methods had certain inhibitory effects on the growth of *C. versicolor*. Under the action of N-CQDs, the diameter of fungal colonies decreased, indicating that N-CQDs have good inhibitory effects. The results of hydrothermal treatment with ethanolamine, urea, and ethanolamine and urea doping into HACC are represented by solutions d (HACC, ethanolamine), e (HACC, urea), and f (HACC, ethanolamine, urea), respectively. Figure S1 shows that the method using solution f was the best nanomodification technology and it led to remarkable antifungal effects, indicating that doping modification was successful. The combination of ethanolamine and urea as dopants during the synthesis of N-CQDs had stronger antibacterial effects than ethanolamine and urea alone and presented synergistic effects [41]. The method using synthetic solution f (HACC hydrothermally modified with ethanolamine and urea) was selected as the optimal doping modification method.

### 2.2. N-CQD Process Optimization Results (Single-Factor and Orthogonal Tests)

The inhibition rate of mycelial growth was used as an evaluation indicator for the experiment. A single-factor experiment with three factors and three levels was conducted on N-CQDs to optimize the synthesis method. Table S1 presents the design of the single-factor experiment on N-CQDs. The N-CQDs presented remarkable antifungal properties when they were synthesized at the m_(HACC)_:m_(urea)_:m_(ethanolamine)_ ratio of 1:2:2, with a time of 8 h and temperature of 180 °C (Figures S2–S4). The single-factor experimental results were used as the basis, and a three-factor, three-level orthogonal experiment was conducted. Table S2 presents the design of the orthogonal experiment on N-CQDs. The orthogonal experiment showed that the N-CQDs prepared at the ratio of m_(HACC)_:m_(urea)_:m_(ethanolamine)_ of 1:2.5:1.5, with a time of 8 h and temperature of 180 °C, had the best *C. versicolor* growth inhibition rate (Tables S3–S5). The yield of the final product was 39.6%, which was higher than the synthesis yield of other carbon quantum dots [42].

### 2.3. Characterization of N-CQDs

**Figure 2:**
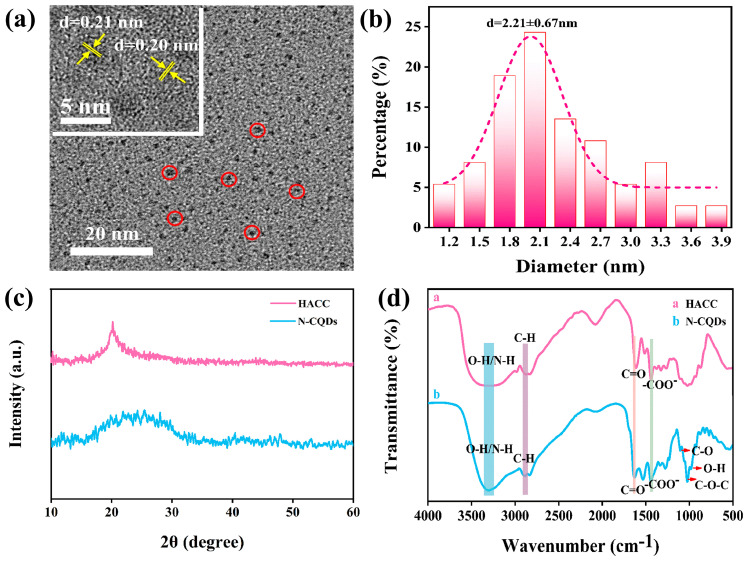
(**a**,**b**) Transmission electron microscope (TEM) and size distribution images of N-CQDs. (**c**) X-ray diffractometer spectra of HACC and N-CQDs. (**d**) Fourier-transform infrared spectroscopy of HACC and N-CQDs at different wavelengths.

The analysis of the morphology and structure of N-CQDs is important. The microstructure of N-CQDs was obtained through TEM (Figure 2a). The synthesized N-CQDs had a size range of 1–5 nm and were all less than 10 nm in size. The particle size was mainly concentrated at 2.21 nm, which conformed to the basic properties of CQDs. No considerable agglomeration was observed, and N-CQDs exhibited a monodisperse oblate shape, indicating that the hydrothermal synthesis of N-CQDs is a simple and effective method. The high-resolution TEM image in the upper left corner shows that N-CQDs had a lattice stripe structure, and its lattice spacing was ~0.21 nm, which corresponds to the (100) crystal plane of graphite (Figure 2a). The particle diameter of N-CQDs was measured and statistically analyzed using a particle size distribution calculation software (Digital Micrograph 3.4) (Figure 2b). The particle size distribution of N-CQDs ranges from 1.15 to 3.85, and the N-CQD size was mainly concentrated at ~2.21 nm, confirming that N-CQDs had small sizes. The N-CQDs’ size is an important feature of their effectiveness as wood preservatives. Nanosized N-CQDs can easily enter wood cell walls, reducing the ability of fungi to degrade wood [43]. 

The XRD images of N-CQDs and HACC are provided in Figure 2c. HACC has an obvious diffraction peak at 2θ = 20.5°, indicating that its crystallinity is very high, which is caused by intramolecular hydrogen bonding, and belongs to the crystallization diffraction of chitosan. The diffraction peak indicates the presence of crystalline regions in HACC; it belongs to the crystal form of cellulose II. After N-CQDs were synthesized using the hydrothermal method, the original crystal diffraction peak of HACC disappeared and the new broad peak appeared at around 26°, and, in the neighborhood of 43°, this diffraction angle corresponds to the (002) and (100) crystal plane of graphite [44], which can be mainly attributed to the graphite carbon structure [45]. This finding, which was consistent with the TEM analysis, showed that HACC was carbonized after the hydrothermal reaction and that a new graphite-like carbon structure formed. The Raman spectrum of N-CQDs exhibited a strong peak at 1512 cm^−1^. The peak was the signal peak of a sp^2^ carbon atom (G-band), which again confirmed the graphitized carbon structure of N-CQDs (Figure S5). The functional groups of N-CQDs and HACC were identified through analyzing absorption peaks and vibration forms. The FT-IR spectra was provided in Figure 2d. HACC and N-CQDs exhibited broad characteristic absorption peaks in the 3000–2850 and 3500–3200 cm^−1^ bands, which were due to the stretching vibrations of C–H and O–H/N–H, respectively. Compared with that of HACC, the peak of N-CQDs at 3256 cm^−1^ had considerably enhanced, demonstrating that N-CQDs had more N–H and O–H groups at 3256 cm^−1^ than HACC, which it may ascribe to the successful doping of nitrogen atoms. The FTIR spectra of N-CQDs and HACC were highly similar, indicating that, after hydrothermal reaction, N-CQDs still retained some of the natural skeletons of HACC. The absorption peaks of HACC and N-CQDs at 1700 cm^−1^ might be due to the stretching vibration of C=O. N-CQDs exhibited an absorption peak at 1402 cm^−1^, which was a characteristic peak generated by the stretching vibration of –COO–. This result showed that N-CQDs contained a large number of –COOH. In addition, N-CQDs exhibited the C–O stretching vibration peaks of alcohol hydroxyl groups at 1097 cm^−1^. The absorption peak at 1047 cm^−1^ was the stretching vibration of C–O–C. An O–H bending peak in the carboxyl group was also found at 945 cm^−1^. FTIR revealed that the synthesized N-CQDs contained abundant functional groups (N–H, C=O, C–H, C–O, O–H, –COOH, and C–O–C) in their structures. The presence of hydrophilic groups enhanced the water solubility of N-CQDs [46]. 

**Figure 3:**
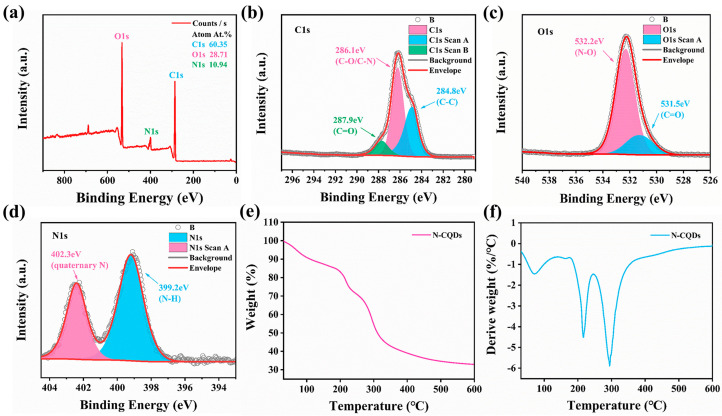
(**a**) X-ray photoelectron spectroscopy (XPS) full spectra of N-CQDs. (**b**) XPS high-resolution C1s spectra of N-CQDs. (**c**) XPS high-resolution N1s spectra. (**d**) XPS high-resolution O1s spectra. (**e**) Thermogravimetric (TG) spectra of N-CQDs. (**f**) Derivative TG spectra of N-CQDs.

The elemental composition and corresponding atomic percentages of N-CQDs were detected through XPS. XPS showed that N-CQDs contained three elements: C, O, and N; the elemental content of the three elements was 60.35%, 28.71%, and 10.94%, respectively (Figure 3a). We performed peak fitting on the elements to analyze their different valence states. Figure 3b presents the XPS spectrum of C1s; it was divided into three peaks at 284.8 eV, 286.1, and 289.7, attributed to C–C, C–O/C–N, and C=O, respectively. The O1s spectrum can be deconvoluted into two peaks at 531.5 eV and 532.2, which were attributable to C=O and N–O, respectively (Figure 3c). In addition, Figure 3d shows the N1s XPS spectrum, which was divided into two peaks located at 399.2 eV and 402.3, was attributable to N–H and quaternary N bonds, respectively [47]. XPS showed that the synthesized N-CQDs contained abundant functional groups (C–C, C=O, N–H, and C–O/C–N).

As shown in Figure 3e,f, we explored the weight changes in N-CQD using a synchronous thermal analyzer at different test temperatures. The combination of TG and derivative thermogravimetry spectra revealed that the weight loss of N-CQDs was divided into three stages with different weight changes. The weight loss during the first stage (30–170 °C) was due to the evaporation of bound water molecules in N-CQDs [26]. The weight loss rate in the second stage (180–250 °C) and the third stage (260–350 °C) was close to 50% and was mainly attributed to the decomposition of carboxyl, hydroxyl, and double bonds in N-CQDs. The weight of N-CQDs gradually decelerated when the temperature exceeded 330 °C until it reached 600 °C, and the N-CQDs retained 30% of their original weight.

**Figure 4:**
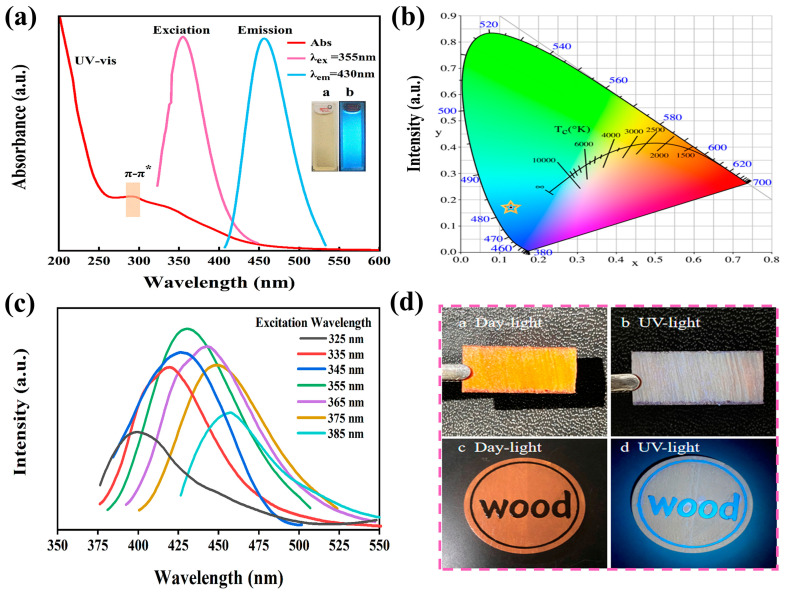
(**a**) UV–vis absorption and PL excitation and emission spectra of N-CQDs in aqueous dispersions (λex = 355 nm, λex = 430 nm), a and b show N-CQD images under natural and ultraviolet light conditions in (**a**). (**b**) Color co-ordinates diagram of N-CQDs. (**c**) Excitation–emission PL spectra of N-CQDs in aqueous dispersions. (**d**) Image of wood blocks impregnated with the N-CQD aqueous solution under natural and ultraviolet light.

The optical characteristics of N-CQDs was analyzed using UV–visible and steady-state fluorescence spectrometers. The UV–visible spectrum of N-CQDs is provided in Figure 4a. N-CQDs exhibited a broad absorption peak at 289 nm that belonged to the π–π * absorption band, which could possibly be ascribe to the transition of C=C or C=O in N-CQDs [48]. Furthermore, the optimal excitation and emission wavelengths of N-CQDs were found at 355 and 430 nm, respectively. Figure 4a presents an image of a N-CQD under natural and ultraviolet light. It shows that N-CQDs appeared light yellow under natural light irradiation and could generate strong blue fluorescence under 365 nm ultraviolet light. N-CQDs exhibited excellent fluorescence properties. The blue fluorescence of N-CQDs was further confirmed by the CIE 1931 standard colorimetric system. The CIE color co-ordinates are (0.1291, 0.1709), as shown in Figure 4b, calculated based on fluorescence emission data at 355 nm. Figure 4c provides the emission spectra of N-CQDs, wavelengths ranging from 325 to 385 nm at intervals of 10 nm. With an increased excitation wavelength, the corresponding maximum emission wavelength gradually red-shifted. Similar to CQDs, N-CQDs exhibited dependence on the excitation wavelength. The N-CQDs prepared by Zhang et al. using chitosan and citric acid as the raw materials exhibited similar characteristics [49]. As shown in Figure 4d, the wood samples were immersed in N-CQD solution (20 mg/mL), cut into thin slices, and exposed to natural and ultraviolet light. The wood blocks appeared dark yellow under natural light and gray under ultraviolet light. N-CQDs exhibited good permeability and fluorescence. Based on this, we used laser technology to print letters on the wood blocks. The N-CQDs aqueous solution was slowly dropped along the dents, and the blue (wood) letters on the wood blocks could be seen under ultraviolet light (365-nm) (Figure 4d). The excellent fluorescence characteristics of N-CQDs can be used to detect their distribution in wood and enable visualization [50].

### 2.4. Antifungal Effects of HACC and N-CQDs

**Figure 5:**
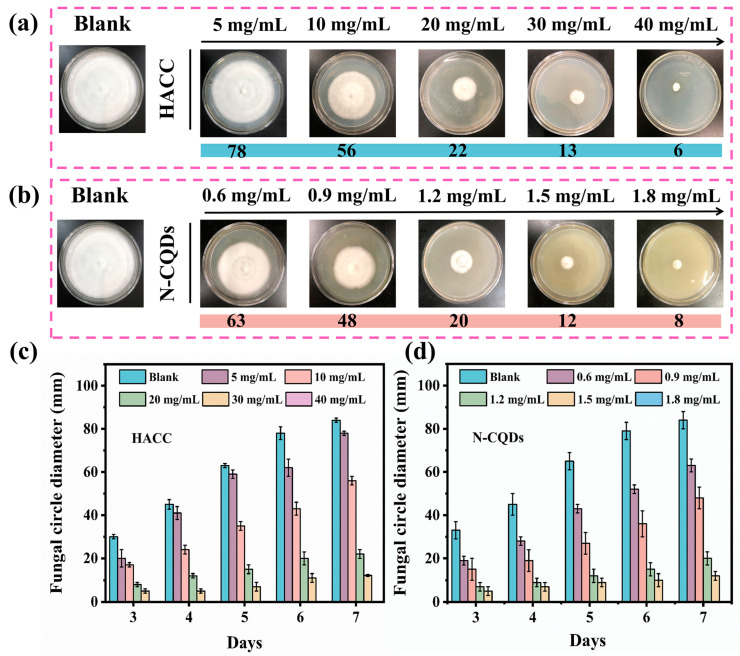
(**a**) Antifungal effect of HACC on *C. versicolor* at 7 days. (**b**) Antifungal effect of N-CQDs on *C. versicolor* at 7 days. (**c**) Colony diameter of *C. versicolor* in the PDA medium with HACC. (**d**) Colony diameter of *C. versicolor* in the PDA medium with N-CQDs.

In this test, we evaluated the antifungal effects of HACC and N-CQDs on a PDA medium, and the antifungal ability of the preservative was determined based on the area of the colonies on the medium for determining their MIC. The culture medium in the blank control group was filled with colonies, indicating that *C. versicolor* had vigorous growth and excellent fungal activity. Therefore, this group could be used as the control group for the MIC test. The antifungal effects of HACC are illustrated in Figure 5a. The antifungal ability of HACC gradually increased with the increase in the preservative concentration. After the N-CQDs were modified with HACC, their MIC value remarkably reduced and their antibacterial effect drastically improved (Figure 5b). The MIC was obtained based on different concentrations of HACC and N-CQDs. The MIC of HACC was 40 mg/mL (Figure 5c), whereas that of N-CQDs was 1.8 mg/mL (Figure 5d). The MIC of N-CQDs was nearly 22 times lower than that of HACC.

### 2.5. Decay Resistance of Wood

**Figure 6:**
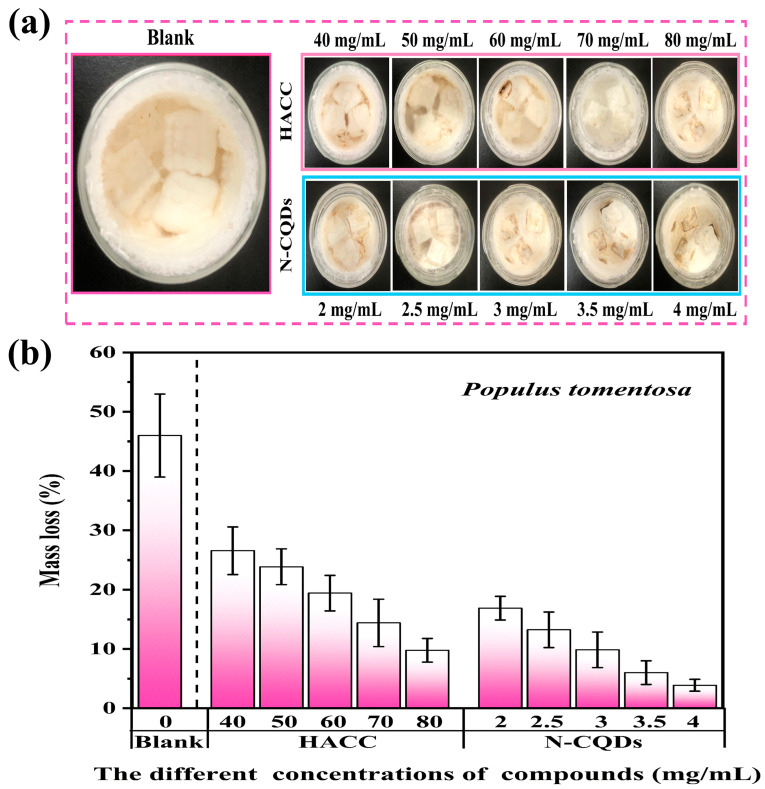
(**a**) Digital photos of the indoor degradation resistance of samples impregnated with different concentrations of HACC and N-CQDs for 12 weeks. (**b**) Mass loss rates of samples impregnated with HACC and N-CQDs.

The evaluation of the wood degradation is according to the mass loss [51]. Decay resistance was divided into four levels, as shown in Table S6. A mass loss rate of 0%–10% is indicative of strong decay resistance and belongs to the strong degradation resistance level. Different concentrations of HACC and N-CQDs were used to treat wood specimens in accordance with MIC values. The wood samples treated with preservatives were exposed to a culture bottle filled with *C. versicolor* for 12 weeks. The results indicated that the trends of the decay resistance of HACC- and N-CQD-treated wood samples were similar to those of MIC (Figure 6a,b and Table S7).

Figure 6a shows that the surface hyphae of the three groups of wood samples proliferated well, indicating that the selected *C. versicolor* strain had sufficient activity. The blank sample surface of the control group has been completely covered with hyphae, whereas the hyphal growth of the preservative-loaded wood block was inhibited. As the concentration of the preservative increased, the surface hyphae of the sample decreased, and the wood sample treated with N-CQDs was more obvious than the wood sample treated with HACC. This finding indicated that the synthesized N-CQDs improved the degradation resistance ability of wood. The mycelia on the surfaces of wood blocks were gently scraped off, and the mass loss rate of the samples after decay was determined. The mass loss in the blank group of wood samples reached 46 ± 0.5% and exceeded 45% (Figure 6b). In accordance with the classification of the wood decay resistance grade, the blank sample lacked decay resistance. The mass loss rate of the sample impregnated with HACC (40 mg/mL) decreased to 26 ± 0.5%. As the concentration of the preservative increased, the antifungal effect was enhanced but remained unsatisfactory. The mass loss rate of wood specimens treated with N-CQDs considerably decreased, and the antifungal effect of the treatment was remarkable. When the concentration was only 3 mg/mL, the mass loss rate of the sample was 9.7 ± 0.5%, reaching the critical value of strong decay resistance. As the concentration of N-CQDs increased, the mass loss rate of the sample gradually decreased. When the concentration was 3.5 mg/mL, the mass loss rate reached 6 ± 0.5%, reaching the strong decay resistance level (Class I). Comparing the wood samples impregnated with N-CQDs and HACC showed that the concentration of N-CQDs was 25 times lower than that of HACC, and N-CQDs had better decay resistance effects; this result was very interesting. The mass loss rate at each concentration was tested five times, and the average value was selected to ensure the accuracy of the experimental data. The comparison between HACC and N-CQDs indicated that HACC modification is necessary and N-CQDs exerted a considerable decay resistance effect when used as a wood preservative.

### 2.6. Inhibitory Mechanism of N-CQDs against C. versicolor

The fungal outer membrane and cell wall are an important component that is responsible for maintaining cell shape, preventing mechanical stress, and maintaining the stability of the intracellular environment. CQDs mainly interact with fungi through electrostatic attraction, functional group modification, and heteroatom doping, causing certain harm to fungi. They can directly act on fungi, causing damage to fungal cell walls and membranes, leading to overall physiological dysfunction. In severe cases, cell contents may leak, ultimately leading to fungal death [52]. The inhibition of the metabolic enzyme activity of microbes can also prevent normal physiological metabolism, resulting in changes in microbial morphology and, ultimately, leading to microbial death [53]. Analyzing the effect of N-CQDs on fungal growth can provide basic guidance for the diversified application of preservatives. Here, we used biological TEM to characterize the morphology and structure of *C. versicolor* before and after adding preservatives and elucidated the antifungal mechanism of N-CQDs against *C. versicolor*.

**Figure 7:**
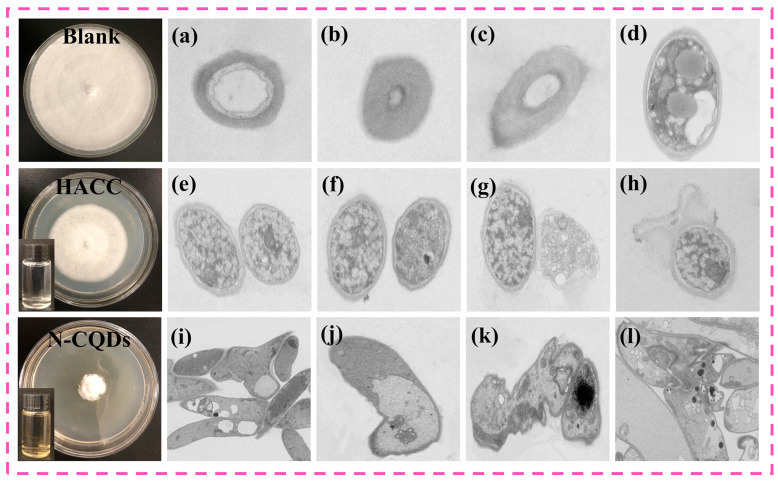
(**a**–**d**) TEM image of the blank group of *Coriolus versicolor*. (**e**–**h**) The image of the *Coriolus versicolor* sample added with HACC. (**i**–**l**) The image of *Coriolus versicolor* added with N-CQDs.

TEM revealed that, in the absence of preservatives, the *C. versicolor* samples exhibited intact structures, presenting a circular or elliptical shape. The cell wall and membrane were not ruptured. For data accuracy, we conducted multiple measurements and found that the cell morphology remained unchanged. *C. versicolor* presented a hollow circular structure that was consistent with the fungal cell structure (Figure 7a–c). In addition, the boundary between the cell membrane and wall was clear, the density inside the membrane was high, and organelles were abundant (Figure 7d). After HACC treatment, the cell morphology and structure of *C. versicolor* showed slight shrinkage, cytoplasm leakage through the cell membrane, organelle disorder, and vesicle disappearance (Figure 7e–h). These effects may be due to the entry of HACC into the fungal cells through their walls and membranes, causing flocculation in the cytoplasm, thereby interfering with the normal cell metabolism and inhibiting the growth of *C. versicolor*. After N-CQD treatment, the cells of *C. versicolor* presented a rod-like shape, causing the cell wall to peel and rupture. A large amount of fungal intercellular content leaked, and the fungal cell structure became severely damaged (Figure 7i–l). This was because N-CQDs often carried positive charges, whereas the plasma membrane and cell wall of fungi were often negatively charged. Therefore, fungi and N-CQDs could undergo electrostatic interactions, leading to changes in cell structure and intercellular content leakage [54]. These effects are important factors for the disruption of fungal cells and inhibition of fungal growth by N-CQDs. In addition, under N-CQD treatment, the organelles in *C. versicolor* disappeared, mainly due to the small-scale effect of N-CQDs. Nanosized N-CQDs are likely to penetrate fungal cells, affecting the activity of fungal metabolic enzymes and inducing fungal cell death. The above experimental results indicated that the antifungal effect of N-CQDs was closely related to cell membrane abnormalities and the in vivo cell enzyme activity of *C. versicolor*.

## 3. Materials and Methods

### 3.1. Materials

HACC (C_6_H_13_NO_4_) was purchased from Yuanye Biotechnology Co., Ltd. (Shanghai, China). Ultrapure water (resistance value: 18 MΩ·cm) was produced by Jingchun Water Treatment Technology Co., Ltd. (Shanghai, China). Urea (CH_4_N_2_O), ethanolamine (C_2_H_7_NO), glucose (C_6_H_12_O_6_), agar (C_12_H_18_O_9_)_n_, sodium dihydrogen phosphate (NaH_2_PO_4_·H_2_O), ethanol (C_2_H_5_OH), glutaraldehyde (C_5_H_8_O_2_), and disodium hydrogen phosphate (Na_2_HPO_4_·H_2_O) were provided by Tianjin Xinbote Chemical Co., Ltd. (Tianjin, China). *C. versicolor* ((L.) *Quel*) was provided by the Mingzhou Biotechnology Co., Ltd. (Ningbo, China) and stored in a 4 °C freezer for future use after activation. Poplar wood (*Populus tomentosa Carr.*, sapwood) was produced from Ulanqab (Inner Mongolia, China). Wood samples were selected from freshly felled poplar wood (2–5 growth rings/cm). The dimensions of the wood blocks were 20 mm × 10 mm × 20 mm (radial × longitudinal × tangential) and 20 mm × 3 mm × 20 mm (radial × longitudinal × tangential). The samples lacked obvious defects, such as knots or cracks, and their air-dried density and moisture content were 0.48 g/cm^3^ and 11.3%, respectively.

### 3.2. Instrumental Analysis

The size and surface morphology of N-CQDs were observed through TEM (Tecnai G2 F20, FEI, Hillsboro, OR, USA). X-ray diffraction (XRD) spectra were recorded using an X-ray diffractometer (D8, Bruker, Karlsruhe, Germany) at a scanning speed of 10°/min and angle range of 5°–60°. The type and chemical structure of N-CQDs were obtained by X-ray photoelectron spectroscopy (XPS) (AXIS Supra, Kratos, Kyoto, Japan) with Al Kα X-ray as the excitation source and a test energy of 1486.68 eV. The weight variation of N-CQDs with increasing temperature was analyzed using a synchronous thermal analyzer (STA200, Hitachi, Tokyo, Japan). The heating rate was 10 °C/min, and the testing temperature of the sample was between 30 °C and 600 °C. The Raman spectra of N-CQDs were obtained through the Raman spectrometer (Invia, Renishaw, London, UK). The UV–visible spectra of N-CQDs were analyzed using a UV–visible spectrophotometer (Lambda 650, Shanghai Yidian Analysis Instrument Co., Ltd., Shanghai, China). The fluorescence characteristics of N-CQDs were analyzed through steady-state fluorescence spectroscopy (F-4600, Hitachi, Tokyo, Japan). The changes in the structure and morphology of *C. versicolor* cells before and after treatment were recorded through biological TEM (H-7650, Hitachi, Tokyo, Japan).

### 3.3. Preparation of N-CQDs and Screening of the Modification Methods

Herein, six types of N-CQDs were synthesized using microwave and hydrothermal methods by doping different nitrogen sources separately (Figure S1). Next, the best modification method was selected based on the antifungal performance of six types of N-CQDs. The specific steps for preparing N-CQDs using microwave and hydrothermal methods are as follows:

Fluorescent N-CQD particles were fabricated using the microwave-assisted method with HACC as the precursor. Specifically, urea (CH_4_N_2_O) and ethanolamine (C_2_H_7_NO) were added to the HACC solution and transferred to a microwave oven for further carbonization to prepare N-CQDs. The microwave power was 700 W, the reaction time was 2 min, and it was repeated three times. The obtained N-CQDs was dissolved in ultrapure water and filtered twice by a circulating water vacuum pump to remove solid impurities. The obtained solution was centrifuged in a centrifuge (7168 RCF, 5 min). Subsequently, the solution was further purified using a filter (0.45 μm). Finally, the above solution was dialyzed with ultrapure water in a dialysis bag (1000-Da) for 48 h, and then dried at a constant temperature (60 °C) to prepare N-CQD powder.

The following modification methods were used during microwave synthesis. a. HACC (0.02 g), ultrapure water (10 mL), and urea were added to a beaker, stirred evenly, ultrasonically treated for 10 min, and then subjected to microwave treatment to synthesize N-CQDs. b. HACC (0.02 g), ultrapure water (10 mL), and ethanolamine were added to a beaker, stirred evenly, treated ultrasonically for 10 min, and then used to initiate N-CQD synthesis through microwave treatment. c. Finally, HACC (0.02 g), ultrapure water (10 mL), urea (0.04 g), and ethanolamine (0.04 g) were added to a bottle, sonicated for 10 min, and then used to start N-CQD synthesis through microwave treatment.

Fluorescent N-CQDs were synthesized using the hydrothermal method with HACC as the precursor. HACC, urea, and ethanolamine were placed in a hydrothermal reactor to prepare N-CQDs. After the high-temperature reaction was completed (180 °C, 6 h), the product was naturally cooled and filtered twice through a circulating water vacuum pump to remove solid impurities. N-CQDs were centrifuged for 5 min (7168 RCF). Subsequently, the obtained suspension was further separated by the filter (0.45 μm). Finally, the N-CQD solution was dialyzed in a dialysis bag (1000 Da) with ultrapure water for 48 h. The product was dried at 60 °C in a drying oven to obtain N-CQD powder.

The following modification methods were used during hydrothermal synthesis. d. Here, 0.02 g of HACC, 10 mL of ultrapure water, and urea (0.04 g) were added to a beaker, mixed evenly, and ultrasonically treated for 10 min. Thereafter, the uniformly stirred solution was added to a hydrothermal reactor to synthesize N-CQDs. e. Then, 0.02 g of HACC, ultrapure water (10 mL), and ethanolamine (0.04 g) were added to a bottle, mixed evenly, and ultrasonically treated (10 min). The solution was placed at hydrothermal reactor to synthesize N-CQDs. f. Finally, 0.04 g of urea, 10 mL of ultrapure water, 0.02 g of HACC, and ethanolamine (0.04 g) were placed in a beaker, mixed evenly, and treated with ultrasound. Then, the treated solution was added to a hydrothermal reactor to prepare N-CQDs. g. The blank group was prepared with 10 mL of deionized water.

N-CQD preservatives synthesized using different nanomodification methods were poured into the potato glucose agar (PDA) medium at the same proportion. The PDA culture medium was cooled, and its surface was inoculated with the mycelia of *C. versicolor*. The Petri dish was sealed with a sealing film and cultured in an incubator (80% RH, 28 °C) for 7 d. The suitable nano-modification technology was determined according to the diameter of the fungal circle.

### 3.4. Optimization of N-CQD Synthesis

N-CQD synthesis with the most effective modification method was further optimized. For the single-factor experiment, the mass ratio of HACC to urea and ethanolamine, treatment temperature, and treatment time were selected as the three factors, and the inhibition rate of mycelial growth was used as the evaluation index. The mass ratio of reactant, treatment time, and treatment temperature on the inhibition rate of mycelial growth were investigated, and an appropriate range was identified. The results of the single-factor experiment were used as the basis and combined with orthogonal experiments to further optimize the synthesis of N-CQDs. N-CQDs prepared under different conditions were added to the PDA medium. After the medium was solidified, *C. versicolor* was inoculated and cultured for 7 d, and the diameter of the fungal circle was recorded. During the test, the PDA medium without preservatives was selected as the blank group, and the growth diameter of fungi was recorded after 7 days (*d*_1_). The fungal circle diameter on the PDA culture medium treated with N-CQDs was determined (*d*_2_). The colony diameter of *C. versicolor* selected during inoculation with the PDA medium was recorded as *d*_3_. The mycelial growth inhibition rate was obtained according to the diameter of the fungal circle using Equation (1):

(1) $$IR=\frac{\left(d_{1}-d_{2}\right)}{\left(d_{1}-d_{3}\right)}\times 100\%$$

where *IR* (%) represents the growth inhibition rate of mycelium after 7 days, *d*_1_ (mm) represents the diameter of the blank colony, *d*_2_ (mm) represents the diameter of the fungal circle on the PDA culture medium after N-CQD addition, and *d*_3_ (mm) represents the colony diameter of *C. versicolor* during inoculation.

### 3.5. Minimum Inhibitory Concentration Test

For the MIC test, firstly, fresh potatoes are peeled and cut into small pieces. Then, 200 g of potato pieces are added to distilled water (1000 mL) and boiled for 15 min. The potato filter residue was filtered out. Further, agar (22 g) and glucose (15 g) were slowly added to the filtrate, and stirred at 80 °C for 10 min. Subsequently, the above culture medium was placed in a high-pressure and high-temperature environment (121 °C, 0.15 MPa) for 30 min. The culture medium was stored after sterilization. Furthermore, HACC solutions (6 mL) with different concentrations (5, 10, 20, 30, and 40 mg/mL) were poured into the above PDA medium (150 mL). A total of 6 mL of N-CQDs solutions of different concentrations (0.2, 0.9, 1.2, 1.5, and 1.8 mg/mL) was synthesized and added to a 150 mL PDA medium. Then, 6 mL of sterilized deionized water was poured into the PDA culture medium as the blank group. Finally, *C. versicolor* was inoculated in solidified PDA medium and cultured in an environment of 28 °C and 80% RH. From Day 3 to Day 7, the growth of hyphae was observed daily and the diameter of colonies was recorded.

### 3.6. In Vivo Test for Determining the Degradation Resistance of Wood

The degradation resistance of wood blocks was determined in accordance with Chinese forestry standards [55]. The blank group and two treatment groups (HACC and N-CQDs) were selected to test wood decay resistance. A total of 165 wood sapwood samples without obvious defects were selected. Among them, 15 untreated wood samples were selected as the blank control group and 75 wood samples impregnated with HACC solution and 75 wood blocks impregnated with N-CQD were selected as the experimental groups. Five concentration gradients were selected for each group, and tests were repeated five times to ensure the reliability of the experiment. In accordance with the minimum inhibitory concentration (MIC) results, the wood blocks were vacuum-immersed in 40, 50, 60, 70, and 80 mg/mL HACC solutions for 30 min and then subjected to the wood decay resistance test. Similarly, five concentrations of N-CQDs (2, 2.5, 3, 3.5, and 4 mg/mL) were used for wood decay resistance testing. First, 105 g of dry river sand, 10.5 g of pine sawdust, 6 g of corn flour, and 0.7 g of brown sugar (0.7 g) were successively placed in a 350 mL wide-mouthed round-capped bottle. Then, three pieces of feed wood were placed in each wide-mouthed round-capped bottle. Maltose solution (70 mL) was slowly added around the feed wood, and the bottle cap was tightened. The cultivation bottle was sealed with a lid; it was continuously sterilized for 1.5 h in a high-pressure steam sterilization pot (1.21 MPa), and then removed and placed in a sterile environment. Subsequently, *C. versicolor* was inoculated in the cooled aforementioned culture medium and cultured for 10 d until the feed wood was completely covered with hyphae. Wood blocks without obvious defects and not treated with preservatives were dried and recorded weight (*m*_1_) at 103 °C. The wood blocks were placed in the above preservative solution and immersed in a vacuum-drying oven for 30 min (relative vacuum degree of −0.09 MPa) to ensure that the preservative was immersed inside the wood. After immersion, the reagent on the surface of the sample was absorbed by absorbent paper, and the weight of the wooden block (*m*_2_) was immediately recorded. Subsequently, the preservative-loaded wood blocks were dried at 60 °C, and their weight was recorded as *m*_3_. Finally, blank wood blocks and preservative-loaded wood were placed on the culture medium filled with mycelium and cultured in a constant-temperature and -humidity (28 °C, 80% RH) incubator for 12 weeks. After the cultivation period, the wood blocks were removed from the incubator, and the mycelium on the surface of the sample was cleaned. The decayed wood samples were dried to constant weight at 103 °C and the weight was recorded (*m*_4_). The loading capacity of the wood blocks for different concentrations of the preservatives was calculated using Equation (2):

(2) $$T=\frac{(m_{2}-m_{1})\times c}{v}\times 10$$

where *T* (kg/m^3^) is the preservative loading of wood sample, *v* (cm^3^) is the sample volume, *m*_1_ (g) is the sample mass without preservative treatment, *m*_2_ (g) is the constant mass of the wood block impregnated with preservatives, and *c* (%) is the preservative concentration.

During the wood degradation test, the mass loss rate was calculated using Equation (3):

(3) $$R=\frac{m_{3}-m_{4}}{m_{3}}\times 100\%$$

where *R* (%) represents the mass loss rate of the wood block, *m*_3_ (g) is the constant mass of the sample treated with preservatives before degradation, and *m*_4_ (g) is the constant mass of the wood block after 12 weeks of degradation testing.

### 3.7. Pretreatment of C. versicolor Specimens

The 2.5% glutaraldehyde solution was prepared for immobilization of *C. versicolor* tissue cells. Biological TEM was used to observe the structural and morphological changes in *C. versicolor* cells. Specifically, 3.16 g of Na_2_HPO_4_·H_2_O was uniformly dispersed in the deionized water (100 mL) to synthesize solution A; 2.76 g of NaH_2_PO_4_·H_2_O was added in deionized water (solution B). Solutions A (61 mL) and B (39 mL) were dispersed evenly to obtain phosphate buffer (0.2 M). Subsequently, phosphoric acid buffer (50 mL, 0.2 M), 10 mL of 25% glutaraldehyde aqueous solution, and deionized water (40 mL) were poured into a bottle and stirred evenly to obtain the 2.5% glutaraldehyde fixing solution, which was stored in a 4 °C freezer. The mycelia of *C. versicolor* along the edge of the blank sample and the colonies under treatment with HACC and N-CQDs were gently scraped off with a cell scraper. The three groups of mycelia were centrifuged for 5 min (1008 RCF). After centrifugation, fungal tissue was collected in a 1.5 mL EP tube. Cell samples were placed in the 2.5% glutaraldehyde fixation solution, dehydrated, embedded, and stained, before being observed under biological TEM at a voltage set to 80 KV.

## 4. Conclusions

N-CQDs with remarkable antifungal effects on *C. versicolor* were synthesized using a one-step hydrothermal method, and their antifungal activity and mechanism were comprehensively studied. Characterization revealed that N-CQDs had abundant functional groups, excellent fluorescence properties, high water solubility, and very small particle sizes. The fluorescence of N-CQDs helped track their distribution in wood. The fungal inhibition results demonstrated that, although HACC and N-CQDs had inhibitory effects on *C. versicolor*, N-CQDs had a more significant antifungal effect. The MIC of HACC was 40 mg/mL, whereas that of N-CQDs was 1.8 mg/mL. The modification method effectively reduced the MIC, and the MIC of N-CQD was ~22 times lower than that of HACC. The decay resistance test results showed that, after N-CQD treatment, the degree of wood decay considerably decreased and the mass loss rate decreased from 46 (±0.5)% to 3.8 (±0.5)%. Research on antifungal mechanisms showed that N-CQDs could damage cell membranes and walls, inhibit metabolic enzyme activity, and lead to fungal death. Therefore, N-CQDs could be considered candidates for developing efficient and sustainable antifungal preservatives. This study provides important ideas for the rational use of carbon nanomaterials to inhibit the fungal decay of wood.

## Data Availability

The data are contained within the article and Supplementary Materials.

## Supplementary Materials

The following are available online at https://www.mdpi.com/article/10.3390/molecules29051013/s1, Figure S1: Antifungal results of 6 different N-CQDs against *C. versicolor*; Figure S2: Different ratio of raw materials for antifungal properties of N-CQDs; Figure S3: Effect of hydrothermal temperature on antifungal activity of N-CQDs; Figure S4: Effect of hydrothermal time on antifungal activity of N-CQDs; Figure S5. Raman images of N-CQDs; Table S1: Single-factor experimental design table; Table S2: Orthogonal test factors—horizontal design table; Table S3: Orthogonal test data table; Table S4: Analysis of variance; Table S5: Range analysis table; Table S6: Evaluation standard for decay resistance of wood; Table S7: Decay resistance of wood treated with different concentrations of HACC and N-CQDs.
