# Supplementary material for: Construction Strategy and Mechanism of a Novel Wood Preservative with Excellent Antifungal Effects
Source: Molecules. 2024 Feb 26;29(5):1013. doi: 10.3390/molecules29051013 (PMC10933868; doi:10.3390/molecules29051013)
Supplement: Supplementary file 1 [file molecules-29-01013-s001.zip › molecules-2884769-supplementary.pdf]

Supporting Information

Construction Strategy and Mechanism of a Novel Wood Preservative with Excellent Antifungal Effects

Lei Wang [a, b, c], Teng Wang [a, c], Ruidi Hao [a], Yamei Wang [a, b, *]

[a] College of Materials Science and Art Design, Inner Mongolia Agricultural University, Hohhot, China;

[b] Inner Mongolia Key Laboratory of Sandy Shrubs Fibrosis and Energy Development and Utilization, Hohhot 010018, P. R. China;

[c] These two authors contributed to this work equally.

*Correspondence: wangym80@126.com (Yamei Wang)

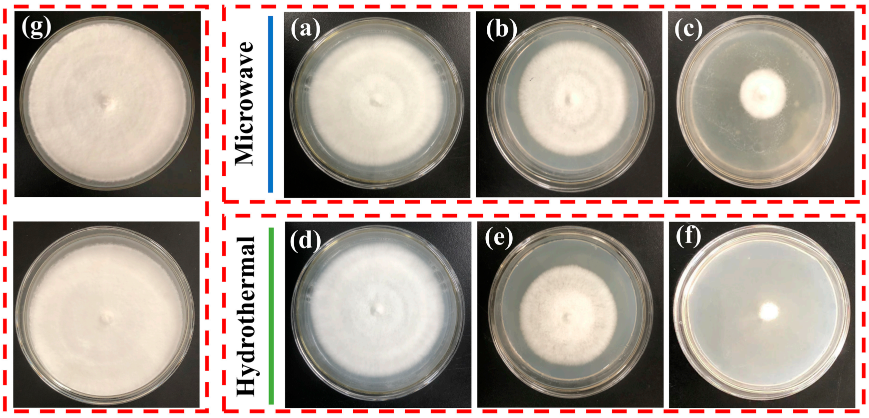

a. Microwave -modified HACC (0.02g HACC + 0.04 g CH_4N_2O +10 mL Ultra-pure water)
b. Microwave -modified HACC (0.02g HACC + 0.04 g C_2H_7NO +10 mL Ultra-pure water)
c. Microwave -modified HACC (0.02g HACC + 0.04 g CH_4N_2O + 0.04 g C_2H_7NO +10 mL Ultra-pure water)
d. Hydrothermal-modified HACC (0.02g HACC + 0.04 g CH_4N_2O +10 mL Ultra-pure water)
e. Hydrothermal-modified HACC (0.02g HACC + 0.04 g C_2H_7NO +10 mL Ultra-pure water)
f. Hydrothermal-modified HACC (0.02g HACC + 0.04 g CH_4N_2O + 0.04 g C_2H_7NO +10 mL Ultra-pure water)
g. Blank group (10 mL Ultra-pure water)

Figure S1. Antifungal results of 6 different N-CQDs against *C. versicolor*.

Tables S1. Single-factor experimental design table.

Serial number	Element		
	$m_{(HACC)}$:$m_{(urea)}$:$m_{(ethanolamine)}$(g)	Temperature (°C)	Time(h)
1	1：0.5：3.5	120	2
2	1：1：3	140	4
3	1：2：2	180	8
4	1：3：1	220	12

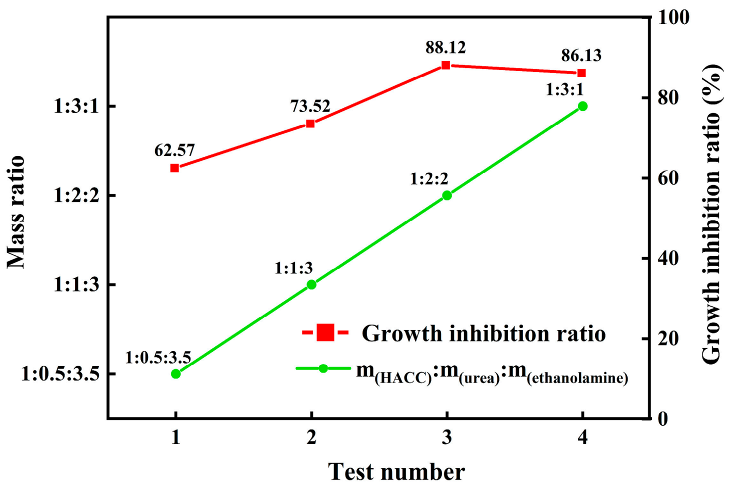

Figure S2. Different ratio of raw materials for antifungal properties of N-CQDs.

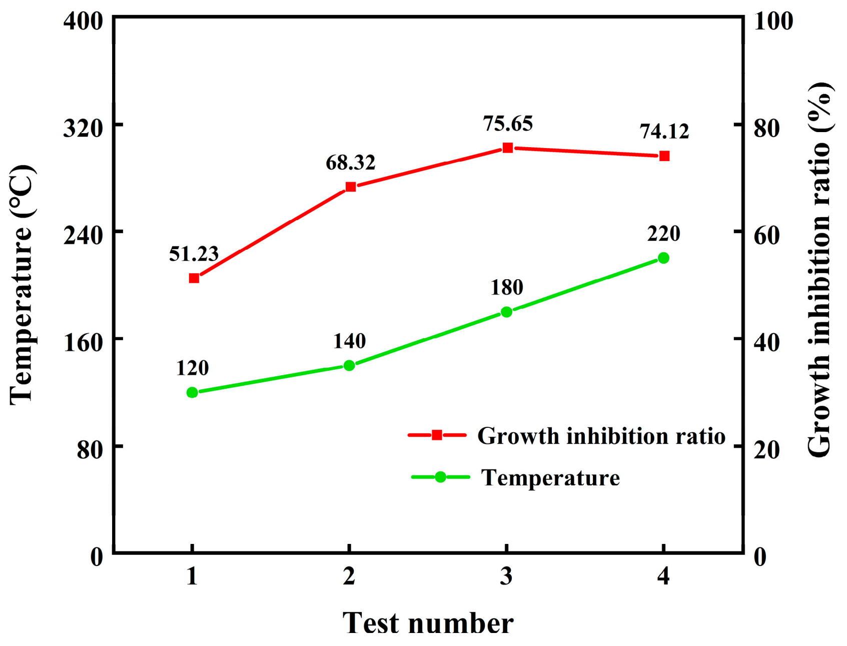

Figure S3. Effect of hydrothermal temperature on antifungal activity of N-CQDs.

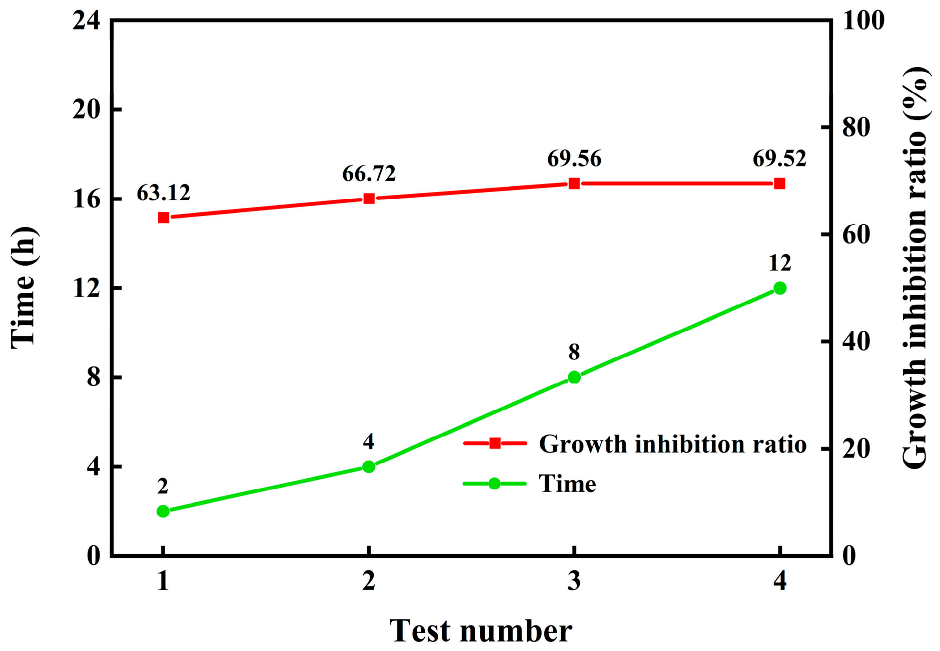

Figure S4. Effect of hydrothermal time on antifungal activity of N-CQDs.

Tables S2. Orthogonal test factors - horizontal design table.

Level	Element		
	$m_{(HACC)}$:$m_{(urea)}$:$m_{(ethanolamine)}$(g)	Temperature (°C)	Time(h)
1	1:1.5:2.5	160	6
2	1:2:2	180	8
3	1:2.5:1.5	200	10

Tables S3. Orthogonal test data table.

Test number	Element / Level			Growth inhibition ratio (%)
	A(g)	B(℃)	C(h)	
1	1	1	1	81.58
2	1	2	2	83.29
3	1	3	3	80.23
4	2	1	2	89.31
5	2	2	3	91.74
6	2	3	1	90.35
7	3	1	3	93.63
8	3	2	1	94.41
9	3	3	2	93.81

Tables S4. Analysis of variance.

Element	Deviation sum of squares	Degree of freedom	F ratio	Significance
A(g)	237.791	2	2.932	*
B(℃)	5.423	2	0.067	
C(h)	0.105	2	0.001	
Error	243.32	6		

Tables S5. Range analysis table.

Test number	A	B	C
K1	81.730	88.173	88.780
K2	90.467	89.813	88.803
K3	93.950	88.160	88.563
Range R	12.220	1.653	0.240
Primary and secondary factors		A＞B＞C	

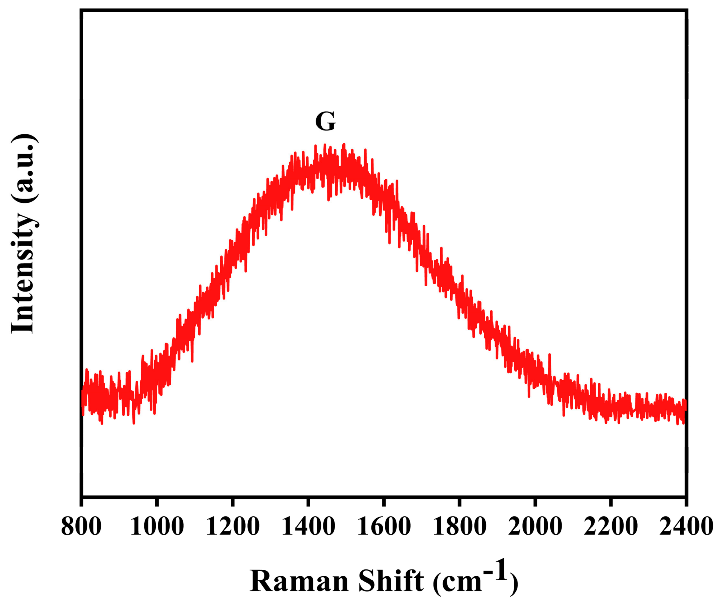

Figure S5. Raman images of N-CQDs.

Tables S6. Evaluation standard for decay resistance of wood.

Rank class	Decay resistance	Mass loss rate/%
I	High decay resistance	0-10
II	Decay resistance	11-24
III	Slight decay resistance,	25-44
IV	No decay resistance	>45

Table. S7. Decay resistance of wood treated with different concentrations of HACC and N-CQDs.

Preservative	Concentration (mg/mL)	Ret (kg/m^3)	Decay resistance grade
HACC	40	21.39	III
	50	26.35	II
	60	30.68	II
	70	35.31	II
	80	39.46	I
N-CQDs	2	1.25	II
	2.5	1.53	II
	3	1.93	I
	3.5	2.35	I
	4	2.47	I
Blank	—	—	IV
